# *OpenStructure*: an integrated software framework for computational structural biology

**DOI:** 10.1107/S0907444913007051

**Published:** 2013-04-19

**Authors:** M. Biasini, T. Schmidt, S. Bienert, V. Mariani, G. Studer, J. Haas, N. Johner, A. D. Schenk, A. Philippsen, T. Schwede

**Affiliations:** a Biozentrum Universität Basel, University of Basel, Klingelbergstrasse 50-70, 4056 Basel, Switzerland; b SIB Swiss Institute of Bioinformatics, Basel, Switzerland; c Department of Physiology and Biophysics, Weill Medical College of Cornell University, New York, NY 10065, USA; d Department of Cell Biology, Harvard Medical School, 240 Longwood Avenue, Boston, MA 02115, USA

**Keywords:** *OpenStructure*, computational structural biology

## Abstract

Research projects in structural biology increasingly rely on combinations of heterogeneous sources of information, *e.g.* evolutionary information from multiple sequence alignments, experimental evidence in the form of density maps and proximity constraints from proteomics experiments. The *OpenStructure* software framework, which allows the seamless integration of information of different origin, has previously been introduced. The software consists of C++ libraries which are fully accessible from the Python programming language. Additionally, the framework provides a sophisticated graphics module that interactively displays molecular structures and density maps in three dimensions. In this work, the latest developments in the *OpenStructure* framework are outlined. The extensive capabilities of the framework will be illustrated using short code examples that show how information from molecular-structure coordinates can be combined with sequence data and/or density maps. The framework has been released under the LGPL version 3 license and is available for download from http://www.openstructure.org.

## Abstract 2

Current developments in the computational structural biology framework *OpenStructure* are presented.

## 1. Introduction

In computational structural biology there is a growing demand for tools operating at the interface of theoretical modelling, X-­ray crystallography, electron microscopy, nuclear magnetic resonance and other sources of information for the spatial arrangement of macromolecular systems (Biasini *et al.*, 2010 ▶; Kohlbacher & Lenhof, 2000 ▶; Philippsen *et al.*, 2007 ▶). Synergy between these fields has led to methods which, for example, combine electron-density information with evolutionary information from sequence alignments and structural information from computational models (DiMaio *et al.*, 2009 ▶; Leaver-Fay *et al.*, 2011 ▶; Alber *et al.*, 2007 ▶; Trabuco *et al.*, 2008 ▶; Rigden *et al.*, 2008 ▶). The need to combine heterogeneous data in incompatible formats is often found to be the reason why new methods in computational structural biology rely on custom-made *ad hoc* combinations of command-line tools built to perform specific tasks. Hence, to facilitate these inconvenient data conversions and to make the development of new methods more efficient, we have developed *OpenStructure* as a powerful and flexible platform for methods development in computational structural biology (Biasini *et al.*, 2010 ▶). This open-source framework provides an expressive application programming interface (API) and seamlessly integrates with external tools, for example for structural superposition and comparison (Zhang & Skolnick, 2005 ▶; Zemla, 2003 ▶; Holm & Sander, 1993 ▶; Olechnovic *et al.*, 2012 ▶), secondary-structure assignment (Kabsch & Sander, 1983 ▶) and homology detection (Altschul *et al.*, 1990 ▶, 1997 ▶; Söding, 2005 ▶). *OpenStructure* has consistently been extended and its API has matured to allow the building of complex software stacks such as homology-modelling software, structure-comparison methods (Mariani, Kiefer *et al.*, 2011 ▶) and model-quality estimation packages (Benkert *et al.*, 2011 ▶).

Since the previous paper on *OpenStructure*, substantial improvements to the graphics, the performance and the user interface have been made and support for molecular-dynamics trajectories, integration of external software tools and data handling has significantly been extended. Here, we first briefly describe the architecture of the *OpenStructure* framework at the code level. We then present the main components of the 1.3 release and the individual modules that interact with molecular structures, density maps and sequence data. Code examples will be used to demonstrate the smooth integration of the *OpenStructure* components.^1^

## 2. Architecture

*OpenStructure* was conceived as a scientific programming environment for computational protein structure bioinformatics with the reuse of components in mind. The functionality of *OpenStructure* is divided into modules dealing with specific types of data. *mol* and *mol.alg* are concerned with molecular structures and the manipulation thereof. *conop* is mainly concerned with the connectivity and topology of molecules. *seq* and *seq.alg* handle sequence data (alignments and single sequences). *img* and *img.alg* implement classes and algorithms for density maps and images. File input and output operations for all data types are collected in the *io* module. *gfx* provides functionality to visualize protein structures, density maps and three-dimensional primitives. *gui* implements the graphical user interface.

The framework offers three tiers of access, in which at the lowest level the functionality of the framework is implemented as a set of C++ classes and functions meeting both the requirement for computational efficiency and low memory consumption. The framework makes heavy use of open-source libraries, including FFTW for fast Fourier transforms (Frigo & Johnson, 2005 ▶), Eigen for linear algebra (v.2; http://eigen.tuxfamily.org) and Qt for the graphical user interface.

The middle layer is formed by Python modules, which are amenable to interactive work and scripting. This hybrid compiled/interpreted environment combines the best of both worlds: high performance for compute-intensive algorithms and flexibility when prototyping or developing applications. In fact, this approach to multi-language computing has found favour with many in the scientific computing community (Schroeder *et al.*, 2004 ▶; Kohlbacher & Lenhof, 2000 ▶; Adams *et al.*, 2011 ▶) and Python has established itself as the *de facto* standard scripting language for scientific frameworks. In addition to general-purpose libraries, *e.g. NumPy* (Dubois *et al.*, 1996 ▶), *SciPy* (http://www.scipy.org/) and the plotting framework *matplotlib* (Hunter, 2007 ▶), there are many bio­informatics and structural biology toolkits that are either completely implemented in Python or provide a well maintained Python wrapper to their functionality (Sukumaran & Holder, 2010 ▶; Chaudhury *et al.*, 2010 ▶; Hinsen & Sadron, 2000 ▶; Kohlbacher & Lenhof, 2000 ▶; Adams *et al.*, 2011 ▶). The combination of general-purpose frameworks with specialized libraries allows new algorithms to be developed with very little effort.

At the highest level, we offer a graphical user interface with three-dimensional rendering capabilities and controls to manipulate structures or change rendering parameters. The three-layer architecture is one of the main strengths of *OpenStructure* and sets it apart from other commonly used tools in computational structure bioinformatics. Rapid prototyping can be performed in Python, and if successful the code can subsequently be translated to C++ for better performance. Since most of the functions in Python have a C++ counterpart, the Python/C++ adaption is straightforward and can be completed in a very short time.

## 3. Molecular structures

**Figure 1:**
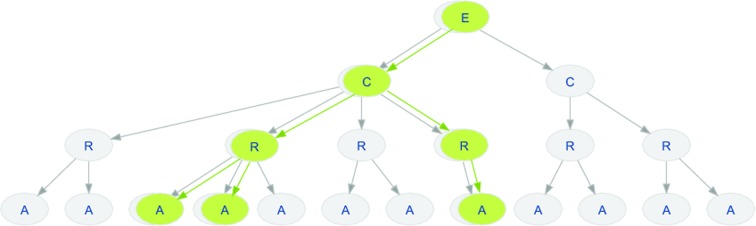
Schematic diagram of the components of entity handles and views. The molecular structure is represented as a tree-like structure rooted at the entity (E). The levels of the tree are formed by chain (C), residue (R) and atom (A). In green, an example entity view containing only a selected subset of elements is shown. The hierarchy of the entity view is separate from the handle; however, at every level the view maps back to its handle, giving access to its properties.

The software module *mol* implements data structures to work with molecular data sets. At its heart lie the EntityHandle and EntityView classes, which represent molecular structures such as proteins, DNA, RNA and small molecules. Other classes deal with molecular surfaces as generated by *MSMS* (Sanner *et al.*, 1996 ▶) or other external tools. The EntityHandle class represents a molecular structure. The interface of entities is tailored to biological macromolecules, but this does not prevent it being used for any kind of molecule: for example, an entity may also represent a ligand or a collection of water molecules, hence the rather generic name. An entity is in general formed by one or more chains of residues. These residues in a chain may be ordered, for example in a polypeptide, or unordered, for example a collection of ligands. A residue consists of one or more atoms. The atoms store the atomic position, chemical element type, anisotropic *B* factor, occupancy, charge, atom bond list *etc.* The hierarchy of chains, residues and atoms is arranged in a tree-like structure rooted at the entity (Fig. 1 ▶). The atoms of an entity may be connected by bonds, which group the atoms of the entity into one or more connected components.

### 3.1. Working with subsets of molecular structures

The processing and visualization of molecular entities often requires filtering using certain criteria. The results of these filtering operations are modelled as so-called EntityViews (Fig. 1 ▶), which contain subsets of atoms, residues, chains and bonds of the respective EntityHandle. The entity view references the original data; for example, modifications to atom positions in the original entity handle are also reflected in the entity view. This handle/view concept pertains to the full structural hierarchy, *i.e.* residue views will only contain the atoms that were part of the filtering *etc.* The EntityView class shares a common interface with the EntityHandle class that it points to and hence they can be used interchangeably in Python. In C++, where type requirements are strict, we employ the visitor pattern (Gamma *et al.*, 1995 ▶) to walk through the chain, residue and atom hierarchy without having to resort to compile-time polymorphism through templates.

The use of entity views throughout the framework makes the implemented algorithms more versatile. For example, the code used to superpose two structures based on C^α^ atoms can also be used to superpose the side chains of a binding site. The only difference is the view and thus the set of atoms that are passed to the superposition function. These sets of atoms do not need to be consecutive and thus arbitrary sets of atoms can be superposed.

### 3.2. The query language: making selections

Entity views are conveniently created by using a dedicated mini-language. Filtering a structure and returning subsets of atoms, residues, chains and bonds is achieved by predicates which are combined with Boolean logic, often referred to as ‘selection’. Typical examples include selecting all backbone atoms of arginines, binding-site residues, ligands or solvent molecules. Conceptually, the language is similar to the selection capabilities of other software packages, *e.g.* *VMD* (Humphrey *et al.*, 1996 ▶), *Coot* (Emsley *et al.*, 2010 ▶) or *PyMOL* (Schrodinger).

The predicates may use any of the available built-in properties defined for the atoms, residues and chains. Examples include the atom name, residue number, chain name or atom element. A complete list of built-in properties is given in the *OpenStructure* documentation. In addition, the predicates may refer to user-defined properties declared using generic properties (see below). The within operator of the query language allows the selection of atoms in proximity to another atom or another previously performed selection.

Since selection statements can be applied both to EntityHandles and EntityViews, complex selections can be carried out by chaining selection statements. For rare cases of highly complex selections, the user may assemble the view manually, for example by looping over the atoms and including atoms meeting some conditions.

### 3.3. Selection example: superposition

As an example of how entity views make *OpenStructure* functions more versatile, we will now consider the binding sites of two haem-containing proteins. We will use the Superpose function of the *mol.alg* module to calculate rotation and translation operators that superpose the atoms of two structures, firstly based on the coordinates of the haem ligands and secondly on the residues binding the haem:
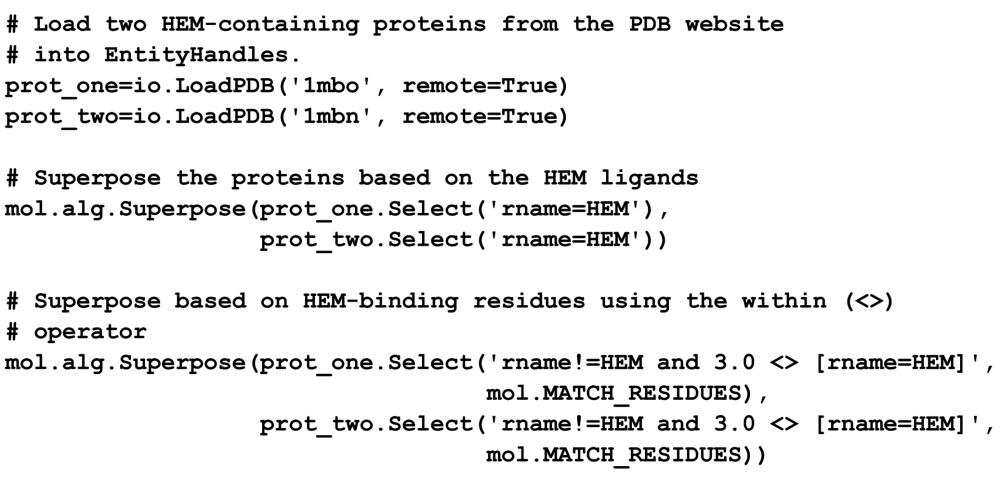
As can be seen, the superposition based on haem atoms or haem-binding residues use the same Superpose function. The only difference is the selection statement to prepare the subset of atoms used to superpose.

### 3.4. Mapping user-defined properties on molecular structures

Many algorithms calculate properties for atoms, residues, chains, bonds or entities. Examples of such properties include the sequence conservation of a residue or local structural similarity scores. *OpenStructure* includes a system to store these properties as key-value pairs in the respective handle classes: the generic properties. Classes deriving from GenericPropertyContainer inherit the ability to store properties of string, float, int and bool type, identified by a key. For each of these data types, methods to retrieve and store values are available in both Python and C++. As with all other built-in properties, the view counterpart will reflect the generic properties of the handle. Since generic properties are implemented at a low level of the API, they are accessible by the query language and may for example be used for substructure selection or in colouring operations.

### 3.5. Connectivity and topology

The *conop* module interprets the topology and connectivity of proteins, polynucleotides and small molecules. For example, after importing a structure from a PDB entry, bonds between atoms have to be inferred and missing information has to be completed. In addition, the *conop* module provides an infrastructure for consistency checks. *OpenStructure* supports two conceptually related yet different approaches for deriving the connectivity information: a rule-based approach that connects atoms based on rules outlined in a database and a heuristic approach which uses a distance-based heuristic.

The rule-based approach to connectivity derivation is based on a set of rules that define the bonding partners for each atom based on its name. The rules are extracted from the chemical component dictionary provided by the wwPDB (Berman *et al.*, 2003 ▶) and are stored in a compound library. Since this library has knowledge of all residues deposited in the PDB, deviations from the rules are easily detected and may be reported back to the user. For automatic processing pipelines that operate on large set of structures, strict settings when loading a structure are advised in order to limit surprises.

For structures from other sources, including molecular-dynamics simulations and virtual screening studies with loosely defined naming conventions, the heuristic approach might be more appropriate. The heuristic builder uses lookup tables for the connectivity of standard nucleotides and standard amino acids, but falls back to a distance-based connection routine for unknown residues or additional atoms that are present in the structure. In contrast to the rule-based approach outlined above, the heuristic builder is meant to be used as a quick-and-dirty connectivity algorithm when working with structures interactively.

### 3.6. Loading and saving molecular structures

*OpenStructure* contains the *io* module for importing and exporting structures from and to various file formats such as PDB, CRD and PQR. In the following, reading of molecular structures and molecular-dynamics trajectory files is described in more detail.

File input is concerned with data from external sources. As such, importers are exposed to files of varying quality. For automated processing scripts, it is crucial to detect non­conforming files during import, as every nonconforming file is a potential source of errors. For visualization purposes and interactive work, on the other hand, one would like files to load, even if they do not completely conform to standards. To account for these two different scenarios, *OpenStructure* introduced IO profiles in v.1.1. A profile aggregates flags that fine-tune the behaviour of both the *io* and *conop* modules during the import of molecular structures. The currently active IO profile controls the behaviour of the importer upon encountering an issue. By default, the import aborts upon encountering a nonconforming file. This strict profile has been shown to work well for files from the wwPDB archive. Many files that could not be loaded using the strict settings, exposed actual problems in the deposited files. These issues have been reported and resolved in the meantime by the wwPDB.

Molecular-dynamics simulations generate a series of coordinate snapshots of the simulated molecule. These snapshots are often stored in binary files. *OpenStructure* supports the reading of CHARMM-formatted DCD files in two different ways. Firstly, the whole trajectory may be loaded into memory. This is the recommended behaviour for small preprocessed trajectories. However, since trajectories may well be larger than the available RAM, loading the complete trajectory is not always an option. The second alternative is to load only a set of frames into memory. The remaining frames are transparently fetched from disc when required. This allows the efficient processing of very large trajectories without consuming huge amounts of memory.

## 4. Sequences and alignments

Since the sequence and the structure of a protein are intrinsically linked, scientific questions in computational structural biology often require the combination of structural and sequence data. In fact, for many applications, methods based on evolutionary information considerably outperform physics-based approaches (Kryshtafovych *et al.*, 2011 ▶; Mariani, Kiefer *et al.*, 2011 ▶). Thus, efficient and convenient mapping between sequence information and the structural features of a protein is crucial.

In *OpenStructure*, the functionality for working with sequences, and the integration with structure data, is implemented in the *seq* module. The principal classes SequenceHandle, AlignmentHandle and SequenceList represent the three most common types of sequence data. Instances of SequenceHandle hold a single, possibly gapped, nucleotide or protein sequence. These instances serve as a container for the raw one-letter-code sequence with additional methods geared towards common sequence-manipulation tasks. The SequenceList is suited for lists of sequences, *e.g.* sequences resulting from a database search using *BLAST* (Altschul *et al.*, 1990 ▶). An AlignmentHandle holds a list of sequences which are related by a multiple sequence alignment. The interface for alignments is focused on column-wise manipulation; for example, the insertion or removal of blocks or single columns. The import of alignments and sequences is supported for the FASTA, *ClustalW* or PIR formats, while export of sequence-related data is implemented for the FASTA and PIR formats.

### 4.1. Efficient mapping of structure-based and sequence-based information

The combination of structure and sequence information is embedded into the core of the SequenceHandle class. A structure may be linked to its matching amino-acid sequence by simply attaching it, defining a relation between information associated with residues in the structure and information related to residues in the sequence. To determine the index of the residue in the protein sequence at the *n*th position in the alignment, the number of gaps prior to *n* needs to be subtracted. A naive mapping implementation counting the number of gaps prior to position *n* would scale linearly with *n*, which is suboptimal for long sequences. For efficiency, the sequence handle maintains a list of all of the gaps present in the sequence. Instead of traversing the complete sequence, traversal of the gap list yields the number of gaps before a certain position. Since the number of gaps is usually much smaller than the sequence length, a more efficient run time is thus observed when mapping between residue index and position in the alignment.

### 4.2. Algorithms for sequences and alignments

The *seq.alg* module contains several general-purpose sequence algorithms. To align two sequences using a local or a global scoring scheme, the Smith–Waterman (Smith & Waterman, 1981 ▶) and Needleman–Wunsch (Needleman & Wunsch, 1970 ▶) dynamic programming algorithms have been implemented. Conservation of columns in an alignment may be calculated with a variation of the algorithm from *ConSurf* (Armon *et al.*, 2001 ▶), which considers the pairwise physico-chemical similarity of residues in each alignment column (for an example, see Biasini *et al.*, 2010 ▶). More sophisticated sequence and alignment algorithms are available through one of the available interfaces to external sequence-search tools such as *BLAST* (Altschul *et al.*, 1997 ▶, 1990 ▶), *ClustalW* (Larkin *et al.*, 2007 ▶), *kClust* (A. Hauser, unpublished work) or *HHsearch* (Söding, 2005 ▶).

### 4.3. Example: ligand-binding site annotation

The following example illustrates how the annotation of ligand-interacting residues for a protein may be automatically inferred from a related protein structure.

Dengue fever is a neglected tropical disease caused by a positive-sense RNA virus which contains a type 1 cap structure at its 5′ end. The dengue virus methyltransferase is responsible for cap formation and is essential for viral replication (Egloff *et al.*, 2002 ▶). Thus, it is an attractive drug target. Four closely related dengue-virus serotypes (DENV1–4) have been isolated; each serotype is sufficiently different such that no cross-protection occurs (Halstead, 2007 ▶). The structure of DENV2 methyltransferase (PDB entry 1r6a; Benarroch *et al.*, 2004 ▶) binds *S*-adenysyl-l-homocysteine (SAH) and ribavirin monophosphate (RVP) in two distinct binding sites. RVP is a weak inhibitor of the activity of the enzyme (Benarroch *et al.*, 2004 ▶). In the structure of DENV3 methyltransferase (PDB entry 3p97; Lim *et al.*, 2011 ▶) only the SAH-binding site is occupied. We would now like to identify which residues in the second structure potentially interact with RVP. Since the two structures share a sequence identity of 77% with each other, the two sequences can be aligned with high confidence using a pairwise sequence-alignment algorithm. Using the mapping defined by the sequence alignment, we then transfer the ligand-binding site information from the first structure to the second structure:
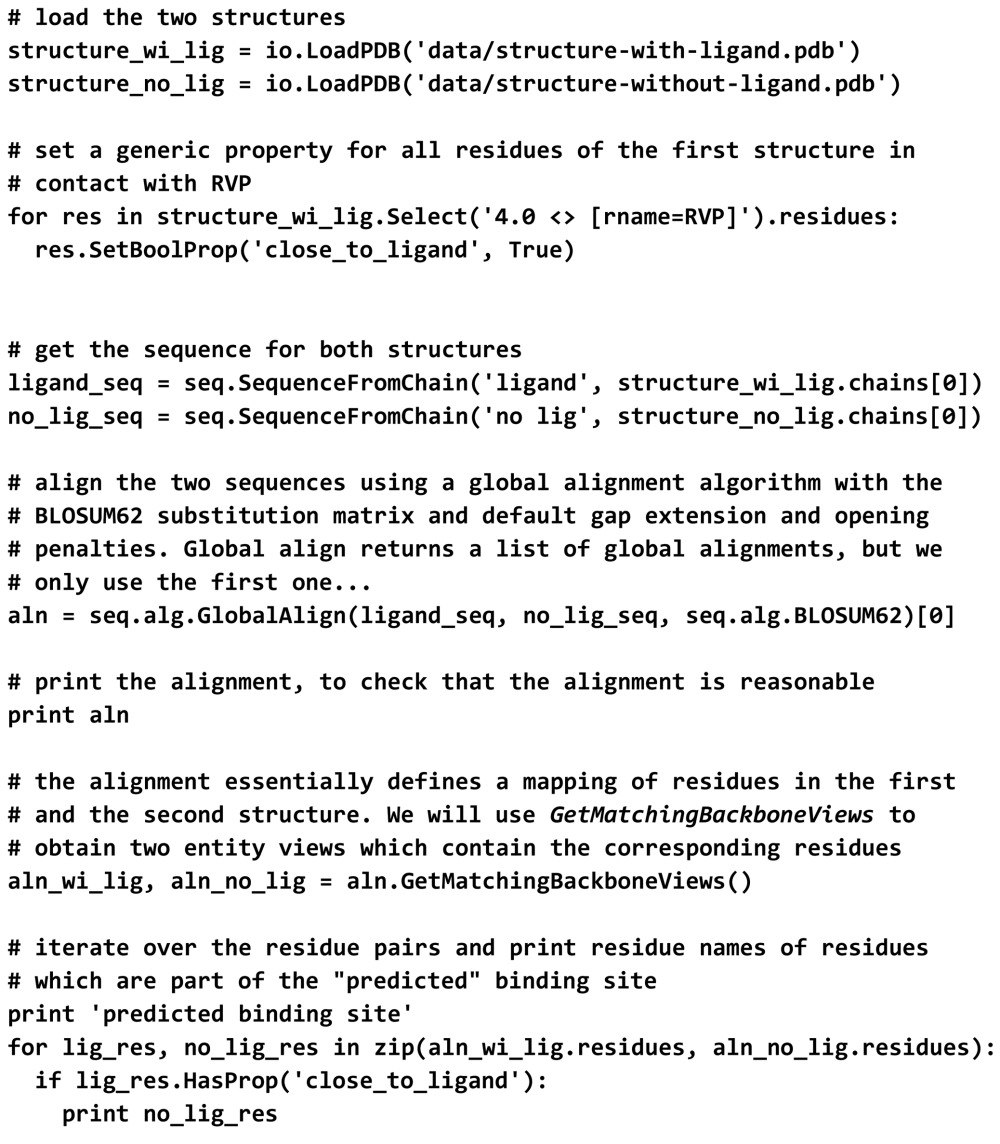


This example illustrates how little effort it takes to map between information contained in two distinct structures. The results are visualized in Fig. 3(*b*). Often, useful scripts can be built with only a few lines of descriptive *OpenStructure* Python code.

## 5. Density maps and images

The majority of available experimental protein structures have been determined using X-ray crystallography. This technique produces density maps into which an atomistic or semi-atomistic model is built. For high-resolution structures, model building into density maps is completely automated (Adams *et al.*, 2011 ▶; Langer *et al.*, 2008 ▶). However, at low resolution automated approaches usually fail and manual intervention is required. As has been repeatedly shown, the integration of theoretical modelling techniques is often able to improve the models built (DiMaio *et al.*, 2009 ▶; Trabuco *et al.*, 2008 ▶). The theoretical modelling field, on the other hand, can profit from the availability of density maps, even at low resolution, to refine homology models.

To provide efficient and convenient access to density data, *OpenStructure* includes the *img* and *img.alg* modules. The core functionality of these two modules was initially developed as part of the *Image Processing Library and Toolkit* (*IPLT*; Philippsen *et al.*, 2003 ▶, 2007 ▶; Mariani, Schenk *et al.*, 2011 ▶). The *IPLT* package implements a complete processing pipeline to obtain density maps from recorded electron micrographs. As part of a joint effort to lower the maintenance burden for the two packages, the core data structures and algorithms of *IPLT* have been moved into *OpenStructure*. The two modules offer extensive processing capabilities for one-, two- and three-dimensional image data. In this module electron-density maps are considered as three-dimensional images, and hence the terms image and density map are used interchangeably.

The principal class of image-processing capabilities is the ImageHandle. It provides an abstraction on top of the raw pixel buffers and keeps track of pixel sampling, dimension and data domain. An ImageHandle can store an image either in real or reciprocal space. The image is aware of the currently active domain. This means, for example, that one can apply a Fourier transformation (FT) to an ImageHandle containing a spatial image and the image will correctly identify the new active domain as frequency. The ImageHandle also supports the application of a FT to an image with conjugate symmetry, resulting in a real spatial image, while applying a FT to an image without conjugate symmetry results in a complex spatial image.

Image and density data may be imported and exported from and to PNG, TIFF, JPK, CCP4, MRC, DM3 and DX files. Standard processing capabilities for images are provided in the *img.alg* module. This module contains filters; for example, low- and high-pass filters, masking algorithms and algorithms to apply a Gaussian blur to an image. Additionally, the module contains algorithms to calculate density maps from molecular structures either in real space or Fourier space (DiMaio *et al.*, 2009 ▶), which we will use in the following example.

### 5.1. Correlating backbone fragments with local electron density

**Figure 2:**
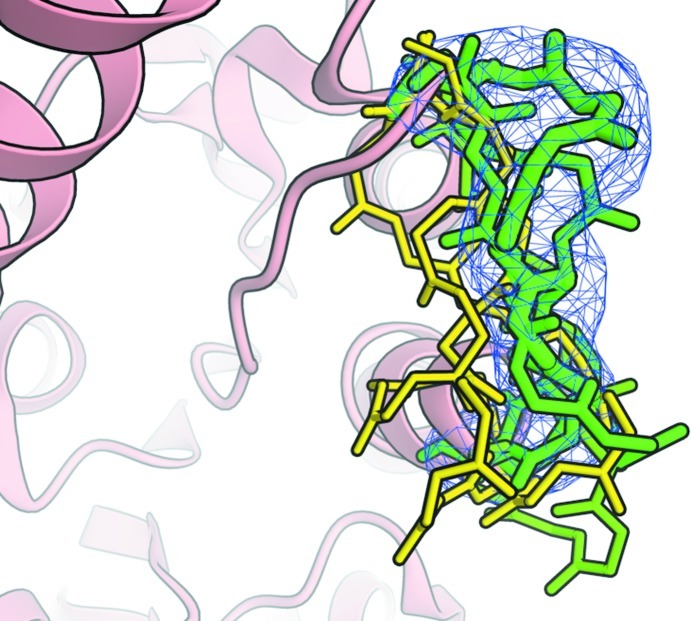
A selection of possible backbone conformations to bridge a fragmented chain. The fragments are coloured by correlation with the density from yellow to green. The tube thickness used to render the backbone fragment is scaled according to the density correlation.

We would like to illustrate the combined use of density maps and structure data in *OpenStructure* in the following paragraph. As an example, consider a protein structure in which a segment of six residues has not been resolved. However, close inspection of the density map reveals that there is substantial experimental evidence to connect the two parts of the protein chain. We would now like to rebuild the missing part of the backbone. Possible conformations are sampled from a database of structurally non-redundant fragments compiled from the PDB. For scoring, the density for the fragment is calculated by placing a Gaussian sphere at the position of every atom. The resulting density map is then compared with the experimental density by real spatial cross-correlation. Fig. 2 ▶ shows a few selected backbone conformations coloured by correlation to the density map.
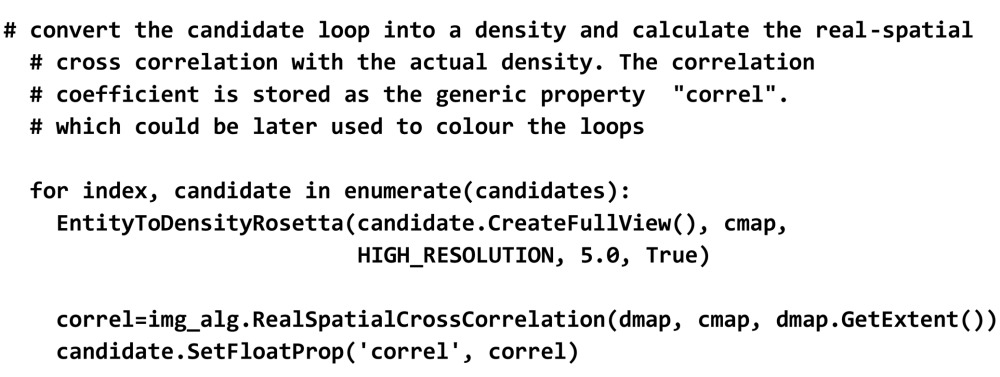


## 6. Visualization

Solutions to challenging scientific and algorithmic problems often become obvious after an appropriate form to display the information has been found. Readily available visualization tools are an enabling factor both for science and algorithm development. *OpenStructure* features sophisticated visualization capabilities as part of the *gfx* module. The rendering engine is capable of producing publication-ready graphics. It has been used for the visualization of very long molecular-dynamics simulations (Yang *et al.*, 2012 ▶; Shan *et al.*, 2012 ▶).

Each principal class of the *mol* and *img* modules has a renderer (graphical object) in the graphics module that is responsible for converting the abstract data into a three-dimensional rendering and supports the display of molecular structures, surfaces and density data. The separation of graphical objects from their corresponding counterparts keeps the orthogonal concepts of display and general manipulation/querying of structural data separate and saves memory when no visualization is required. Graphical objects are organized by scene: a scene graph-like object. The scene manages the currently active graphical objects and is responsible for rendering them. In addition, the scene manages rendering parameters such as light, fog, clipping planes and camera position.

The rendering engine has been implemented with OpenGL. Typically, each of the graphical objects calculates the geometry, *i.e.* the vertices and triangle indices, once and stores it in vertex buffers. Since the geometry of most objects does not change in every frame, storing the geometry allows more efficient rendering of large structures. If possible, the vertex buffers are transferred to the video memory of the graphics cards to save the round-trip time of sending the geometry over the system bus. For advanced effects, the *gfx* module uses the OpenGL shading language (GLSL). The fixed pipeline shaders of OpenGL are replaced by custom shaders which implement special lighting models, *e.g.* cartoon or hemi-light shading, shadows or ambient occlusion effects.

**Figure 3:**
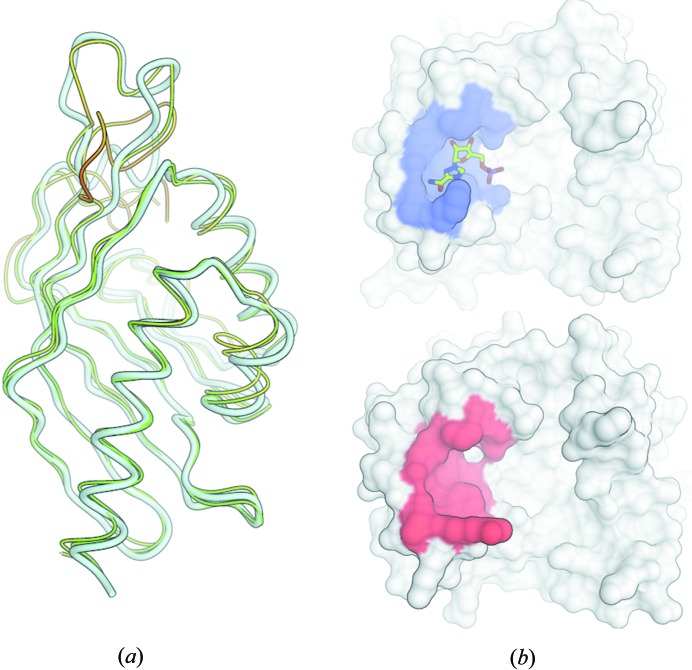
Two distinct visualization styles illustrating the graphical capabilities (see text for a more detailed description). (*a*) Hemi-light shading with outline mode, (*b*) simplified enzyme representation by its molecular surface together with an inhibitor.

Fig. 3 ▶ shows two images generated with the graphics module of *OpenStructure*. The scripts to generate these images have been deposited as Supplementary Material. Fig. 3 ▶(*a*) is inspired by a recent analysis of modelling performance within the *Continuous Automated Model EvaluatiOn* assessment framework (*CAMEO*; http://www.cameo3d.org). The target structure is shown in tube representation (white colour, larger tube radius) together with two theoretical models (thin tubes). The models are coloured with a traffic-light gradient from red to yellow to green using a superposition-free all-atom structural similarity measure called the local distance difference test (lDDT; Mariani, Kiefer *et al.*, 2011 ▶). The combination of outline render mode with hemi-light shading gives this image a very clear style. Fig. 3 ▶(*b*) shows the structures of the methyltransferases of two different dengue virus serotypes as described in the example in §[Sec sec4.3]4.3. At the top the enzyme is in complex with the inhibitor ribavarin monophosphate, whereas at the bottom no ligand is present in this binding pocket. The enzyme is represented by its molecular surface as calculated by *MSMS* (Sanner *et al.*, 1996 ▶) and the inhibitor is shown in stick representation. The surface of the observed (top) or the predicted residues (bottom) interacting with the ligand are highlighted in blue or red, respectively.

### 6.1. Visual data-exploration example: proteomics cross-links

The following example illustrates how the visualization of structure-based predictions can help to rationalize the planning of proteomics cross-linking experiments. Large macromolecular structures are difficult to crystallize and often only diffract to limited resolution where it is unfeasible to determine the structure in atomic detail. It is thus common practice to solve the structure of individual components separately and to use other experimental techniques to identity the relative orientations of the components. Proteomics cross-links are one such experimental technique (Leitner *et al.*, 2010 ▶), in which isotope-labelled cross-linkers such as disuccinimidyl glutarate (DSG) or disuccinimidyl suberate (DSS) are added to the sample. The cross-linking reaction chemically connects primary amines, *i.e.* the terminal amines of lysine side chains, which are in close proximity. After protein digestion with trypsin, the cross-linked fragments are identified using mass spectrometry.

**Figure 4:**
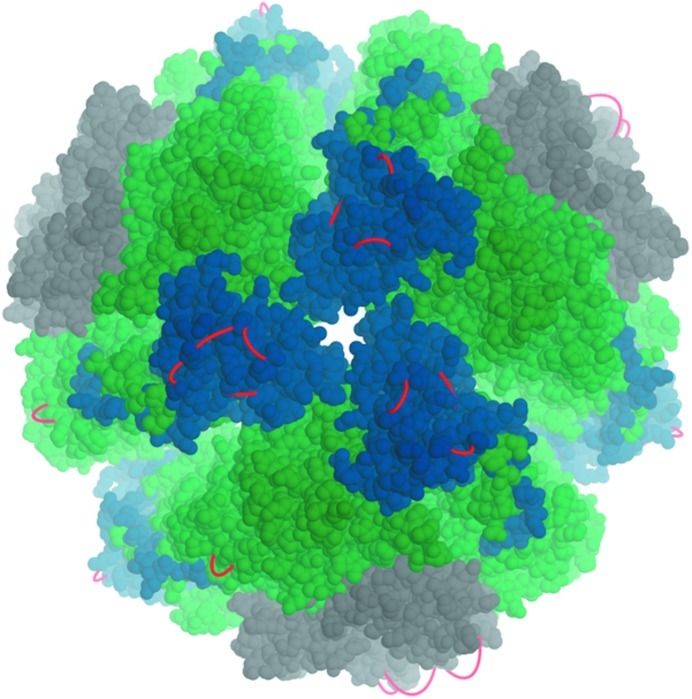
Visualization of predicted cross-link locations in a homology model of the urease from *Y. enterocolitica*. The subunits of the urease are coloured blue (α subunits), green (β subunits) and grey (γ subunits).

Urease from *Yersinia enterocolitica* is a large oligomeric complex that is vital to the pathogenicity of the bacterium. The enzyme catalyzes the cleavage of urea to ammonia at the expense of protons in order to reduce the acidity during the bacterium’s passage through the stomach. To investigate which cross-links are theoretically possible for this protein, we have built a homology model based on the X-ray structure of the urease from *Helicobacter pylori* (PDB entry 1e9y; Ha *et al.*, 2001 ▶), which shares 50% sequence identity. Possible cross-linking sites were then identified using *Xwalk* (Kahraman *et al.*, 2011 ▶). Visualizing the cross-links by connecting the lysine atoms by a straight line does not lead to conclusive results, as the straight lines pass through the protein. To overcome this visualization problem, we used *OpenStructure* to simulate the cross-links as strings of beads. By introducing a force that drives the beads away from the centre of the protein, their positions are optimized. The cross-links appear as red loops sticking out from the surface of the protein. The resulting image (Fig. 4 ▶) of the proteomics cross-links is visually appealing and easily conveys the message that all connections represent intra-chain, not inter-chain, cross-links.

The efficient visualization of the expected outcome allows effective planning of experiments, in this case indicating that experimental proteomics cross-linking data will not contain sufficient information to determine the relative orientation and stoichiometry of the components of the urease complex. The *OpenStructure* script used to generate the example is given in the Supplementary Material.

### 6.2. Graphical user interface

**Figure 5:**
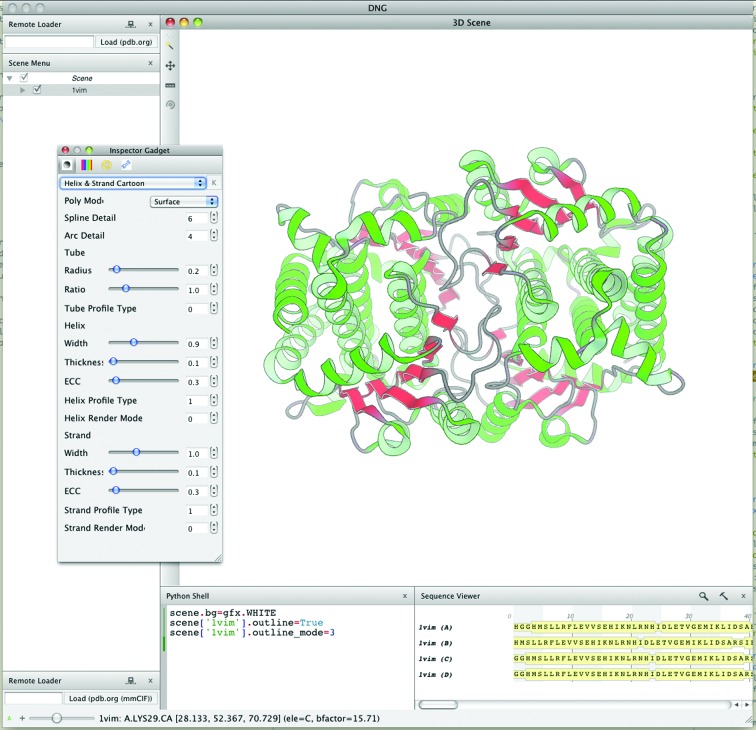
Screenshot of the graphical user interface *DNG*. Controls for data display are organized in a main application window. By default, the majority of the main window is taken up by the three-dimensional scene window, which shows a structure rendered in ribbon mode. The user interacts with the scene using the mouse and keyboard shortcuts. On the left side the currently loaded graphical objects are shown in the scene as a tree view that reflects the structure in the scene graph. The render parameters of graphical objects may be changed using the inspector widget displayed on top of the three-dimensional window. In the bottom right corner the sequences of the loaded proteins are shown.

For interactive work, we have developed a graphical user interface called *DNG* (*DINO*/*DeepView Next Generation*; Guex *et al.*, 2009 ▶). This graphical user interface builds on the visualization and data-processing capabilities of the *OpenStructure* framework and provides controls to interact with macromolecular structures, sequence data and density maps (Fig. 5 ▶). A central part of DNG is the Python shell, which allows efficient prototyping and interaction with the loaded data at runtime. Objects may be queried, modified and displayed using the *OpenStructure* API. For convenience, the shell supports tab-completion and multi-block editing: complete functions and loops may be pasted into the Python shell.

## 7. Conclusions

*OpenStructure* is a software framework tailored towards computational structural biology. Its modular and layered architecture makes it an ideal platform for hypothesis-driven research and methods development, particularly when density maps, molecular structures and sequence data are to be combined. Together with powerful visualization capabilities, the expressive API allows new algorithms to be implemented in a very short time. Additionally, through a variety of bindings, third-party applications can be included into the scripts without worrying about input and output file formats.

*OpenStructure* has been successfully used as an analysis and development platform in several recently published research projects, *e.g.* *QMEAN* (Benkert *et al.*, 2011 ▶), the local difference distance test (Mariani, Kiefer *et al.*, 2011 ▶), the identification of two-histidine one-carboxylate binding motifs in proteins amenable to facial coordination to metals (Amrein *et al.*, 2012 ▶; Schmidt *et al.*, 2011 ▶), the evaluation of template-based modelling (Mariani, Kiefer *et al.*, 2011 ▶), the assessment of ligand-binding site prediction servers (Schmidt *et al.*, 2011 ▶) and the visualization of very long molecular-dynamics simulations (Yang *et al.*, 2012 ▶; Shan *et al.*, 2012 ▶).

## Supplementary Material

OpenStructure scripts.. DOI: 10.1107/S0907444913007051/ic5090sup1.zip

Supplementary material file. DOI: 10.1107/S0907444913007051/ic5090sup2.txt

## Footnotes

1 Code examples and other supplementary materials have been deposited in the IUCr electronic archive (Reference: IC5090). Services for accessing this material are described at the back of the journal.
